# Evaluation of data quality of interRAI assessments in home and community care

**DOI:** 10.1186/s12911-017-0547-9

**Published:** 2017-10-30

**Authors:** Sophie E. Hogeveen, Jonathan Chen, John P. Hirdes

**Affiliations:** School of Public Health and Health Systems, University of Waterloo, 200 University Ave W, Waterloo, ON N2L 3G1 Canada

**Keywords:** interRAI, RAI-HC, Resident Assessment Instrument – Home care, interRAI CHA, Community Health Assessment, Assessment, Quality

## Abstract

**Background:**

The aim of this project is to describe the quality of assessment data regularly collected in home and community, with techniques adapted from an evaluation of the quality of long-term care data in Canada.

**Methods:**

Data collected using the Resident Assessment Instrument – Home Care (RAI-HC) in Ontario and British Columbia (BC) as well as the interRAI Community Health Assessment (CHA) in Ontario were analyzed using descriptive statistics, Pearson’s r correlation, and Cronbach’s alpha in order to assess trends in population characteristics, convergent validity, and scale reliability.

**Results:**

Results indicate that RAI-HC data from Ontario and BC behave in a consistent manner, with stable trends in internal consistency providing evidence of good reliability (alpha values range from 0.72-0.94, depending on the scale and province). The associations between various scales, such as those reflecting functional status and cognition, were found to be as expected and stable over time within each setting (r values range from 0.42-0.45 in Ontario and 0.41-0.43 in BC). These trends in convergent validity demonstrate that constructs in the data behave as they should, providing evidence of good data quality. In most cases, CHA data quality matches that of RAI-HC data quality and shows evidence of good validity and reliability. The findings are comparable to the findings observed in the evaluation of data from the long-term care sector.

**Conclusions:**

Despite an increasingly complex client population in the home and community care sectors, the results from this work indicate that data collected using the RAI-HC and the CHA are of an overall quality that may be trusted when used to inform decision-making at the organizational- or policy-level. High quality data and information are vital when used to inform steps taken to improve quality of care and enhance quality of life. This work also provides evidence that a method used to evaluate the quality of data obtained in the long-term care setting may be used to evaluate the quality of data obtained through community-based measures.

**Electronic supplementary material:**

The online version of this article (10.1186/s12911-017-0547-9) contains supplementary material, which is available to authorized users.

## Background

In order to appropriately inform health care decisions, data at the individual and population levels must be of high quality. Many types of quality problems can affect health care data (see Hirdes et al. [1], for a detailed overview), including error (random and systematic), inappropriate auto-population, incompleteness, and logical inconsistencies [2–5]. Random error is an inherent part of all health care data reflecting chance variations that result in a disagreement between observed and “true” scores of the individual being assessed. Depending on its extent, this type of error may make it difficult to detect true differences between populations or to identify relationships between variables. Systematic error may occur intentionally or unintentionally, and it may lead to incorrect conclusions about the true nature of the relationships between variables of interest [6].

Another threat to the quality of assessment data is the practice of using prior records to automatically complete an assessment without further examination of the person’s current status based on other more up to date sources of information. The effect of such auto-population can be to negate detection of true change in the person’s health, potentially masking evidence of the impact of care provided. This is an especially important problem for longitudinal quality indicators based on rates of improvement or decline in health status [7, 8] since autopopulation will falsely inflate the rates of no change in the population considered. Missing values and coding inconsistencies leading to logical errors are further concerns as they may make observations unusable, thereby decreasing sample size.

Hirdes et al. [1] elaborated on an earlier method used by Phillips and Morris [9] to evaluate the quality of data obtained with the Resident Assessment Instrument - Minimum Data Set 2.0 (RAI-MDS 2.0) in the Continuing Care Reporting System, managed by the Canadian Institute for Health Information (CIHI, www.cihi.ca). The RAI-MDS 2.0 is mandated for use in complex continuing care hospitals/units in Ontario and in long-term care homes in 9 Canadian provinces and territories (see Table 1 for an overview of assessments and data management systems) [10]. The RAI-MDS 2.0 data were found to be consistently high in terms of reliability, validity, completeness, and have a low rate of logical errors [1]. The methods used to analyze data quality for those facilities could likely also be used to examine data from other health sectors even if the specific measures and associations examined differed based on the clinical profiles of the populations being considered.

**Table 1:** Overview of relevant assessments

	RAI-MDS 2.0 (Resident Assessment Instrument – Minimum Dataset 2.0)	RAI-HC (Resident Assessment Instrument – Home Care)	interRAI HC (interRAI Home Care)	interRAI CHA (interRAI Community Health Assessment)
Purpose	To assess the needs, strengths, and preferences of vulnerable populations			
Applications	Care planning, resource allocation, outcomes measures, and quality indicators			
Setting	Long-term care (LTC), Complex continuing care (CCC)	Home care (HC)	Home care (HC)	Community support services (CSS)
Jurisdiction in Canada	Mandated in 9 provinces and territories	Mandated in 8 provinces and territories	Not yet implemented in Canada	Use encouraged in Ontario, determined at organizational level
Data Repository				
Provincial	None	Ontario Association of Home and Community Care (OACCAC)	n/a	Integrated Assessment Record
National	Continuing Care Reporting System (CCRS)^a^	Home Care Reporting System (HCRS)^a^	n/a	None

^a^Managed by the Canadian Institute for Health Information (CIHI)

In addition to the pan-Canadian use of the RAI-MDS 2.0, eight Canadian provinces and territories have implemented the Resident Assessment Instrument – Home Care (RAI-HC) as the mandated assessment for home care services [10–13]. Numerous papers have reported on the reliability and validity of the RAI-HC and its more recent version referred to as the interRAI Home Care (see, for example, [11, 14–16]). Beyond Canada, there are 10 countries internationally, including the United States, France, and New Zealand, with large-scale implementation of the RAI-HC or interRAI Home Care planned or underway. As with the RAI-MDS 2.0, a number of provincial and national home care data repositories have been established in Canada. The Ontario Association of Community Care Access Centres (known as Health Shared Services Ontario as of 2017) receives and compiles data from each Community Care Access Centre (integrated into the Local Health Integration Networks as of 2017) in the province. Community Care Access Centres are single point entry agencies that use the RAI-HC to evaluate needs, determine service eligibility, develop care plans and contract home care services for long stay home care clients (i.e., persons on service for 60 days or more). The Home Care Reporting System is a national database for the RAI-HC and related assessments managed by the Canadian Institute for Health Information. RAI-HC data in the Home Care Reporting System are submitted to the Canadian Institute for Health Information by organizations in British Columbia, Alberta, Saskatchewan, Manitoba, Ontario, Nova Scotia, Newfoundland and Labrador, and the Yukon. In addition, the Canadian Institute for Health Information supports the implementation of the RAI-HC in First Nations communities in Alberta (see Table 1 for an overview of assessments and data management systems).

The threats to data quality in home care differ somewhat from those in long term care facilities. The absolute number of individuals receiving home care services is greater than those residing in long-term care facilities, but there is also more heterogeneity within and between organizations in the populations being served in home care [7]. This heterogeneity may affect the nature of associations between variables that may be used to assess convergent validity. For example, among clients with dementia, the relationship between cognition and physical function could be different than among clients with cerebral palsy. Further, clients often receive care from multiple providers and agencies and they may be seen at lower frequencies and for shorter durations than would be typical for long term care home residents or post-acute hospital patients. Consequently, there is an increased reliance on self-report measures and informal caregivers are depended on as major informants about the person’s health status. Further, it is difficult to conduct traditional psychometric testing in this setting due to time and resource constraints. For example, in order to assess inter-rater reliability, multiple assessors would have to visit the clients’ home when their schedules are often already overwhelmed.

In contrast to the complex continuing care hospitals, long-term care homes and home care sectors in Canada, there is no standardized reporting system for data collected in the community support services (CSS) sector (see Table 1 for an overview of assessments and data management systems). The interRAI Community Health Assessment (CHA) is an assessment similar to the RAI-HC that is used in Ontario to support clinical decision-making, resource allocation, best practices and quality initiatives for vulnerable adults living in the community [14, 17]. The CHA is typically used for persons with somewhat lighter care needs than home care clients and/or receiving social services in the community. While the CSS sector does serve clients with more complex care needs, these clients are usually also served by the home care sector, and therefore assessed with the RAI-HC by the Community Care Access Centre. In addition, not all CSS organizations use the CHA. As a result, data collected with the CHA generally represent only clients on the lower spectrum of need and do not represent the whole population of clients served by the CSS sector. The CHA is modular in nature, with a core component used with all persons assessed and accompanying supplements that are completed based on the presence of specific problems (functional, mental health, assisted living and deafblind supplements are available). It covers domains such as cognition, communication, mood, functional status, and health conditions, among others.

The CSS sector is very heterogeneous, made up of agencies of various sizes, from small volunteer-run organizations to large multi-service providers [18]. CSS organizations provide a range of home and community care services, including friendly visiting, adult day programs, homemaking, meals on wheels, and community nursing. The use of the CHA is determined at an organizational level. Clients within certain programs designated by each organization have an interRAI Preliminary Screener for Primary and Community Care Settings completed in order to determine need for a core CHA assessment and potential supplements. As described above, if a client also receives home care services, they are assessed by the Community Care Access Centre using the RAI-HC and are generally not also assessed using the CHA. While CSS organizations compile and store their own CHA data, there is no national reporting system in place through the Canadian Institute for Health Information as with the RAI-HC and RAI-MDS 2.0. The Integrated Assessment Record does provide a reporting solution provincially. For those CHA-assessor organizations who upload their assessment records to the Integrated Assessment Record, there is an organization-level report available to them. A data sharing agreement for the CHA is in place between the Ontario Ministry of Health and Long-term Care and researchers at interRAI Canada/University of Waterloo.

The threats to CSS data quality are similar to those in the home care sector, although are heightened by less stringent policies and data management practices around CHA assessments. Further, many CSS organizations do not have the same administrative support for completing assessments, storing data and ensuring quality as the Community Care Access Centres in the home care sector. The same methods that have been used to evaluate the quality of long-term and continuing care data will be used to evaluate the quality of home care data and CSS data.

### Study objectives

The objective of the present study is to determine whether techniques used to evaluate RAI-MDS 2.0 data quality can be adapted and used to monitor RAI-HC data and CHA data. It aims to describe the quality of data collected through the RAI-HC in Ontario from 2003 to 2014 and in British Columbia (BC) from 2008 to 2014. These were selected because they represented the largest and best established RAI-HC data holdings at the time of the study. The present study also aims to describe the quality of CHA data collected in Ontario from 2013 to 2016 in order to determine the potential for using this method for monitoring data quality in the CSS sector.

## Methods

### Data sources

Data for the present study were obtained from four sources: Ontario RAI-HC data from the Ontario Association of Community Care Access Centres; British Columbia (BC) RAI-HC data from the Home Care Reporting System; Ontario RAI-HC data from the Home Care Reporting System, and; CHA data from the Integrated Assessment Record (obtained through the Ontario Ministry of Health and Long-Term Care). An additional table shows the number of assessments included in the time series trend analyses from each setting and province by year (see Additional file 1).

Ontario RAI-HC data from 2003 to 2014 were obtained through the Ontario Association of Community Care Access Centres (*N* = 2,626,133 RAI-HC assessments). This source was used for the majority of analyses to take advantage of the largest data holdings with the widest timespan of RAI-HC assessments in Ontario. Hospital versions of RAI-HC assessments and any assessments without a client identifier were excluded. Assessments were sorted by date and the assessment closest to July 1st per individual per year was retained for analyses, reducing the number of observations to 1,743,218 assessments.

RAI-HC data from BC were obtained through the Home Care Reporting System, for the period of 2009 to 2014 (*N* = 245,101 RAI-HC assessments). Any assessments without a client identifier were excluded. Assessments were sorted by date and the assessment closest to July 1st per individual per year was selected to be included in analyses. The final BC dataset included 208,735 RAI-HC assessments.

In order to further compare data quality in Ontario and BC, data from Ontario were also obtained from the Home Care Reporting System, dating from October 2010 to October 2011. The data for this particular analysis included 1,406,054 assessments from Ontario and 121,343 assessments from BC (also limited to October 2010 to October 2011). The smaller data cuts were used for this comparison analysis to ensure the data from both Ontario and BC were contemporaneous and filtered through the same data filters at the Canadian Institute for Health Information.

CHA data were obtained from the Ontario Ministry of Health and Long-Term Care and include the CHAs uploaded by CSS organizations to the Integrated Assessment Record, which amounted to 56,359 assessments from 2013 to 2016. Not all agencies are tasked with using the CHA if their services are restricted to non-clinical supports. Further, not all CSS organizations using the CHA upload their assessments to the Integrated Assessment Record. As a reminder, if a client also receives home care services, they are assessed by the Community Care Access Centre using the RAI-HC and are generally not also assessed using the CHA. Assessments were sorted by date and the assessment closest to July 1st per individual per year was retained for analyses. Assessments without a client identifier were excluded. The final dataset included 45,179 CHA assessments.

### Variables

Table 2 provides an overview of the variables used in the analyses.

**Table 2:** Description of variables

Abbreviation	Name	Purpose	Range	Cut-off
ADLH	Activities of Daily Living Hierarchy	Categorizes ADL loss according to disablement process	0 (no impairment) – 6 (total dependence)	≥ 3 (Extensive assistance needed to total dependence)
ADL Long Form	Activities of Daily Living – Long Form	Sum of seven items assessing performance of ADLs	0 (independent in all ADLs) – 28 (completely dependent in all ADLs)	n/a
CHESS	Changes in Health, End-Stage Disease, Signs, and Symptoms Scale	Measure of health instability, identifies individuals at risk of serious decline	0 (no at all unstable) – 5 (highly unstable)	n/a
CMI	Case Mix Index	Cost weight value assigned to each RUG-III/HC group that reflects the relative resource use per day of an individual within that group compared to the overall average resource use per day within a specific population	0.45 (cost is 0.45 times that of overall average) – 5.75 (cost is 5.75 times that of overall average)	n/a
CPS	Cognitive Performance Scale	Reflects cognition, based on memory impairment, level of consciousness, and executive function	0 (intact) - 6 (severe impairment)	≥ 3 (moderate to severe impairment)
DRS	Depression Rating Scale	Measures signs and symptoms of depression	0 (no mood symptoms) – 14 (all mood symptoms present in last 3 days)	≥ 3 (presence of symptoms of moderate to severe depression)
IADL Performance Scale	Instrumental Activities of Daily Living Performance Scale	Sum of three items assessing performance of IADLs	0 (independent in all IADLs) – 9 (completely dependent in all IADLs)	≥ 3 (at least some help needed with IADLs)
IADL Capacity Scale	Instrumental Activities of Daily Living Capacity Scale	Sum of three items assessing capacity to perform IADLs	0 (no difficulty to perform all IADLs) – 6 (great difficulty to perform all IADLs)	≥ 3 (some or great difficulty to perform IADLs)
MAPLe	Method for Assigning Priority Levels	Differentiates clients into levels based on risk of adverse outcomes, highly correlated with need for long-term care, caregiver distress, and client considered to be better off living in another setting	0 (self-reliant, no major problems in function, cognition, behaviours or environment) – 5 (presence of ADL impairment, cognitive impairment, wandering, behaviour problems)	≥ 3 (moderate to high risk of long-term care placement and/or caregiver distress)
Pain Scale		Uses two measures of pain (frequency and intensity) to create summary score	0 (no pain) – 3 (daily severe pain)	n/a
RUG-III-HC	Resource Utilization Groups –Home Care	Reflects relative intensity of services and supports a client is likely to use	Assigns clients to groups within categories (44 groups)	n/a

The *Cognitive Performance Scale (CPS)* uses information from items assessing memory impairment, level of consciousness, and executive function to provide a score reflecting cognition. Scores range from 0 (intact) to 6 (very severe impairment). A score of 3 or more indicates moderate to severe impairment. The CPS has been validated against the Mini-Mental State Exam (MMSE) in several studies [16, 19, 20].

The *Depression Rating Scale (DRS)* measures signs and symptoms of depression. This scale was validated against the Hamilton Depression Rating Scale and the Cornell Scale for Depression. Scores range from 0 (no mood symptoms) to 14 (all mood symptoms present in last 3 days). A score of three or more indicates the presence of symptoms of moderate to severe depression [21, 22].

The *Method for Assigning Priority Levels (MAPLe)* is an algorithm that differentiates patients/clients into five priority levels based on their risk of long-term care placement and caregiver distress. Individuals in the lowest priority group are considered self-reliant and do not have any major problems in function, cognition, behaviours, or their environment. The highest priority levels are based on the presence of activities of daily living (ADL) impairment, cognitive impairment, wandering, and behavior problems [23, 24]. In the CHA assessment, results can only be obtained from this algorithm when the functional supplement module is completed.

The *Activities of Daily Living Hierarchy (ADLH)* categorizes ADLs as early, middle, and late loss according to the disablement process in which they occur and assigns them a score accordingly. Early loss ADLs, such as dressing, are assigned lower scores and late loss ADLs, such as eating, are assigned higher scores. Scores range from 0 (no impairment) to 6 (total dependence) [16, 25].

The *Activities of Daily Living (ADL) Long Form* is the sum of seven items assessing performance of ADLs: mobility in bed, transfers, locomotion, dressing, eating, toilet use, and personal hygiene. This scale ranges from 0 to 28, with lower scores indicating more self-sufficiency in performance of ADLs.

The *Instrumental Activities of Daily Living (IADL) Performance Scale* is the sum of three items assessing performance of IADLs: meal preparation, ordinary housework, and phone use. The scale ranges from 0 to 9, with lower scores indicating greater independence and higher scores indicating greater need for assistance in performing IADLs [25]. The *Instrumental Activities of Daily Living (IADL) Capacity Scale* is the sum of three items assessing the real or potential difficulty for a client to perform IADLs: meal preparation, ordinary housework, and phone use. The scale ranges from 0 to 6, with lower scores indicating little difficulty and higher scores indicating great difficulty.

The *Changes in Health, End-Stage Disease, Signs, and Symptoms (CHESS) Scale* is a measure of health instability and identifies individuals at risk of serious decline. This scale has been shown to predict death in the community and in long-term care settings, as well as hospitalization, pain, caregiver distress and poor self-rated health. Scores range from 0 (not at all unstable) to 5 (highly unstable) [15, 26, 27].

The *Pain Scale* uses two measures of pain (frequency and intensity) to create a summary score from 0 (no pain) to 3 (daily severe pain). This scale has been shown to predict pain when validated against the Visual Analogue Scale [25, 28].

The *Resource Utilization Groups – Home Care (RUG-III/HC)* algorithm groups clients into 44 groups reflecting the relative intensity of services and supports they are likely to use. This algorithm explains 33.7% of variance in formal and informal resource use in the home care setting and has been shown to be valid in a Canadian population [29–31]. Clients with lower resource use fall into the categories of reduced physical function while those with higher resource use fall into the categories of special care, extensive services, or special rehabilitation. The *Case Mix Index* is a cost weight value assigned to each group that reflects the relative resource use per day of an individual within a RUG-III/HC group compared to the overall average resource use per day within a specific population.

### Analysis

In the Vancouver Island Health Region of BC data from 2008 to 2014 and in the Vancouver Coastal Health Region of BC data from 2008 to 2012, there is no accurate way to distinguish assessments completed in the hospital. Sensitivity analyses were performed to determine whether the findings from this work would change using different methods to identify likely hospital version assessments. The conclusions remained the same, so all BC RAI-HC assessments were included in order to maximize the number of assessments.

Yearly time series trends were examined to describe population characteristics and the resource intensity of home care and CSS clients over time. Trends in convergent validity were analyzed using Pearson’s r correlations for variables expected to be related to each other where the relationship is likely to be stable over time. The variables included ADL hierarchy and CPS; IADL and CPS; DRS and pain scale; and CHESS and pain scale. This method is based on the approach used in Hirdes et al. [1] in their analysis of the quality of Canadian RAI-MDS 2.0 data in the Continuing Care Reporting System and Phillips and Morris [9] in their analysis of the quality of American Minimum Data Set 2.0 data.

Trends in reliability were assessed using Cronbach’s alpha to measure internal consistency for four parallel form scales that are embedded in the RAI-HC and the CHA: performance of instrumental activities of daily living (IADLs), capacity to perform IADLs, activities of daily living (ADL) – long form, and the depression rating scale (DRS). These four different kinds of scales were selected because they have varying levels of known reliability and the consistency of the reliability across settings at different levels provides information about the quality of data.

In order to assess for potential auto-population, the data were examined for the absence of change in six particular sets of indicators. These indicators included informal hours of care in the past week, ADL function (9 items), IADL performance (7 items), IADL capacity (7 items), IADL performance and capacity combined (14 items), and mood (9 items). If an individual was completely independent, required no informal care, or exhibited none of the mood symptoms at both time points, they were not considered to be cases of auto-population. For the informal care items, the values for the number of hours of weekday informal care and weekend informal care were summed. If the sum value was identical at the first assessment in each year as the second assessment in the same year, auto-population was considered to possibly have occurred. For the ADL, IADL and mood items, if the values were the same at the first assessment in each year as the second assessment in the same year for all items in each domain, auto-population was considered to possibly have occurred. This analysis was only performed with the RAI-HC data from Ontario. In BC, the RAI-HC data were not usable for evaluating auto-population since reassessments are not performed as often as in Ontario. Similarly, reassessments are not often performed in the CSS sector so this evaluation could not be performed with CHA data.

Finally, as a further indicator of data quality, the patterns of associations between numerous variables in the RAI-HC data in Ontario and BC were examined to determine if they were similar in both provinces. The same analysis was conducted comparing Ontario RAI-HC data and Ontario CHA data to determine whether data in the home care sector and CSS sector behaved similarly.

### Ethics clearance

This study was reviewed and received ethics clearance through the Office of Research Ethics (ORE) at the University of Waterloo (ORE#18228 and ORE#19917).

## Results

### Population characteristics

Tables 3 and 4 provide the trends in population characteristics of RAI-HC assessed home care (HC) clients in Ontario (2003-2014) and BC (2008-2014), and CHA-assessed CSS clients in Ontario (2013-2016). Characteristics examined included gender, marital status, age, the percentage of clients with dementia, heart failure, and a CPS, DRS, MAPLe, ADLH, IADL capacity and self-performance score of three or more. These results provide information on the comparability of the populations across settings and over time, while recognizing that the data do not represent all CSS clients or all CHA-assessed clients. In all three samples, the majority of clients were female, but the percentage of Ontario clients who were female declined over time (from 69.5 years in 2003 to 63.9 in 2015). There was also a modest reduction of the percentage of females in BC. There were only about 2% to 3% more females among CHA-assessed CSS clients than found among Ontario and BC HC clients and this percentage remained quite stable over time. In both provinces and all settings, the minority of clients were married. Ontario had a higher percentage of clients under the age of 65 and a lower percentage of clients over the age of 85 than in BC. The CHA data had similar percentages of clients in each age group as Ontario HC clients. There is a higher proportion of clients with dementia in BC than in both Ontario samples. The CHA-assessed CSS clients had the lowest rates of dementia, decreasing slightly over time. The percentage of heart failure clients ranged from 11.5% to 14.4% in Ontario HC clients, and from 14.2% to 14.9% in BC HC clients, although the percentage among CHA-assessed CSS clients was lower. These percentages were quite stable over time in BC, but there is an increase in the percentage of clients with dementia in Ontario HC clients, from 16.0% to 24.2%, and a slight decrease in CHA-assessed CSS clients, from 19.4% to 17%.

**Table 3:** Trends in demographic characteristics

	Female (%)			Married (%)			Under 65 (%)			Over 85 (%)			Dementia (%)			Heart Failure (%)		
Year	ON HC	BC HC	CHA	ON HC	BC HC	CHA	ON HC	BC HC	CHA	ON HC	BC HC	CHA	ON HC	BC HC	CHA	ON HC	BC HC	CHA
2003	69.5			36.3			16.0			28.7			16.0			14.4		
2004	68.6			37.5			16.7			28.2			16.0			13.5		
2005	67.5			38.2			17.9			28.4			15.8			12.8		
2006	66.8			38.4			18.2			29.2			16.1			12.7		
2007	66.4			38.7			18.1			30.3			16.8			12.5		
2008	66.1	64.7		38.3	29.5		17.8	12.0		31.4	40.1		17.3	32.0		11.9	14.2	
2009	65.8	64.3		38.4	28.8		17.6	11.9		32.5	42.6		17.9	33.8		11.6	14.3	
2010	65.3	64.4		38.7	26.6		17.4	11.0		33.6	44.6		19.5	34.9		11.5	14.7	
2011	64.8	63.4		38.8	27.1		17.3	11.5		34.5	44.8		20.4	35.5		11.5	14.8	
2012	64.7	62.9		38.3	28.0		16.4	10.6		36.0	45.7		22.1	37.8		11.7	14.9	
2013	64.2	63.2	66.3	38.0	28.7	24.7	14.9	10.5	17.3	37.7	45.7	34.1	24.1	37.7	19.4	12.1	14.8	7.7
2014	63.9	63.2	66.3	37.8	30.0	26.0	15.0	10.3	17.1	38.2	46.6	35.0	24.2	37.5	17.4	12.2	14.8	8.4
2015			66.8			26.7			16.3			35.0			16.4			7.8
2016			67.9			25.7			13.9			37.2			17.0			8.6
Mean (SD) of annual % rates	66.1 (1.74)	63.7 (0.73)	66.8 (0.75)	38.1 (0.68)	28.4 (1.24)	25.8 (0.83)	16.9 (1.14)	11.1 (0.67)	16.2 (1.55)	32.4 (3.62)	44.3 (2.23)	35.3 (1.34)	18.8 (3.16)	35.6 (2.20)	17.6 (1.31)	12.37 (0.88)	14.65 (0.29)	8.1 (0.51)

*BC HC* British Columbia RAI-HC data from the Home Care Reporting System, *CHA* Community Health Assessments from Ontario, *ON HC* Ontario RAI-HC data from the Ontario Association of Community Care Access Centres

**Table 4:** Trends in clinical characteristics

	CPS ≥ 3 (%)			DRS ≥ 3 (%)			MAPLe ≥ 3 (%)				ADLH ≥ 3 (%)			IADL (cap) ≥ 3 (%)		IADL (perf) ≥ 3 (%)			
Year	ON HC	BC HC	CHA	ON HC	BC HC	CHA	ON HC	BC HC	CHA (All)	CHA (FS)	ON HC	BC HC	CHA	ON HC	BC HC	CHA	ON HC	BC HC	CHA
2003	11.4			12.6			60.6				12.8			70.9			73.0		
2004	11.3			12.1			61.3				12.8			72.3			74.7		
2005	10.6			12.8			61.3				12.0			71.7			75.0		
2006	10.5			14.1			61.4				11.3			70.7			75.5		
2007	10.3			14.1			62.3				11.4			70.6			75.8		
2008	10.1	21.1		13.9	21.8		62.7	82.6			11.3	21.2		70.3	84.6		75.5	83.0	
2009	10.3	21.6		14.0	19.4		63.7	82.8			11.8	19.9		70.8	84.0		76.1	82.8	
2010	11.6	21.3		16.4	19.3		69.0	82.8			13.5	19.2		75.5	83.6		79.2	82.1	
2011	13.1	22.7		18.3	19.6		73.3	83.0			14.7	20.8		78.8	83.3		81.5	81.0	
2012	14.4	24.9		19.7	20.5		77.3	84.6			15.9	22.1		81.9	84.8		83.8	81.4	
2013	15.8	25.3	10.8	21.8	20.7	12.7	82.0	85.0	53.0	74.2	17.9	22.2	15.5	85.1	85.1	66.2	86.4	81.0	63.0
2014	16.0	25.4	9.5	22.9	20.7	11.0	83.7	85.2	50.9	73.2	18.2	21.9	13.7	85.9	85.6	66.0	87.2	81.5	62.5
2015			8.4			10.9			51.1	73.9			12.4			65.7			63.1
2016			7.8			11.9			53.0	73.4			11.8			68.5			65.7
Mean (SD) of annual % rates	12.1 (2.19)	23.2 (1.95)	9.1 (1.32)	16.0 (3.74)	20.3 (0.91)	11.6 (0.85)	68.2 (8.66)	83.7 (1.15)	52.0 (1.18)	73.6 (0.46)	13.6 (2.50)	21.0 (1.17)	13.4 (1.64)	75.4 (5.99)	84.4 (0.82)	66.6 (1.26)	78.3 (4.90)	81.8 (0.83)	63.6 (1.45)

*ADLH ≥ 3* Activities of Daily Living Hierarchy scale score of 3 or more, *BC HC* British Columbia RAI-HC data from the Home Care Reporting System, *CHA* Community Health Assessments from Ontario, CHA (All): Results from all CHA assessments, including those without a completed functional supplement module, which contains this variable, CHA (FS): Results from only CHA assessments with a completed functional supplement module which contains this variable, *CPS ≥ 3* Cognitive Performance Scale of 3 or more, *DRS ≥ 3* Depression Rating Scale score of 3 or more, *IADL (cap) ≥ 3* Instrumental Activities of Daily Living capacity score of 3 or more, *IADL (perf) ≥ 3* Instrumental Activities of Daily Living self-performance score of 3 or more, *MAPLe ≥ 3* Method for Assigning Priority Levels score of 3 or more, *ON HC* Ontario RAI-HC data from the Ontario Association of Community Care Access Centres

Overall, the findings related to clinical characteristics (see Table 4) point to a trend of increasing client complexity in Ontario RAI-HC assessed HC clients and higher (but relatively stable) rates of indicators of complexity in BC. These rates trended down in the Ontario CHA-assessed CSS clients. The percentage of Ontario HC clients with a CPS score of three or more (indicating moderate to severe cognitive impairment) increased from 11.4% to 16.0%, whereas the BC percentages ranged between 21.1% and 25.4% and the CHA-assessed CSS clients percentages decreased from 10.8% to 7.8%.

While there was an increasing proportion of clients with a DRS score of three or more (indicating possible depression) in Ontario over time, the initially higher percentage in BC remained stable around 20%. The percentage of CHA-assessed CSS clients with a DRS score of three or more was lower than in the home care sector, ranging from 10.9% to 12.7%.

The patterns for other clinical indicators tended to follow that of CPS. That is, the percentage of clients who had a MAPLe, ADLH, IADL capacity and IADL self-performance scores of three or more: a) were initially highest in BC HC clients; b) were lowest in CHA-assessed CSS clients; c) rose over time in Ontario HC clients to comparable levels as seen in BC. In other words, the Ontario and BC HC clients became more similar over time, whereas the Ontario CSS clients assessed with the CHA generally had distinctly lower levels of complexity than the HC samples.

Tables 5 and 6 show trends in service utilization and resource intensity, including mean total hours of informal care; the receipt of any physical therapy, occupational therapy, nursing or personal support worker services; and the percentage of clients in the lowest and highest Resource Utilization Groups for Home Care (RUG-III-HC) groups (Reduced Physical Function Pa_1 or Pa_2) and Extensive Services (SE_1 to SE_3). In addition, the mean Case Mix Index (CMI) score based on both formal and informal care is provided. In both Ontario and BC, RAI-HC assessed HC clients received an average of between 18 to 20 h of informal care per week compared with between about 11 to 15 h of informal care for CHA-assessed CSS clients in Ontario.

**Table 5:** Trends in service utilization

	Mean Total Informal Hours of Care			Any PT (%)				Any OT (%)				Any Nursing (%)				Any PSW (%)			
Year	ON HC	BC HC	CHA	ON HC	BC HC	CHA (All)	CHA (FS)	ON HC	BC HC	CHA (All)	CHA (FS)	ON HC	BC HC	CHA (All)	CHA (FS)	ON HC	BC HC	CHA (All)	CHA (FS)
2003	20.6			7.6				5.9				30.5				73.5			
2004	20.0			8.1				6.5				30.0				68.6			
2005	19.5			9.1				7.4				31.6				67.6			
2006	18.9			8.2				7.5				31.3				67.5			
2007	18.3			8.4				8.0				30.5				66.8			
2008	17.9	20.0		8.9	10.6			8.6	7.5			29.2	9.3			67.3	51.6		
2009	17.7	18.2		8.8	9.8			8.9	7.0			27.9	9.5			66.6	54.1		
2010	18.2	19.2		8.9	8.9			9.2	6.5			27.0	11.3			66.1	57.8		
2011	18.5	18.9		10.3	8.6			11.2	6.0			27.9	12.2			68.3	58.9		
2012	18.8	19.5		11.3	8.1			13.0	5.6			26.2	12.1			69.9	58.7		
2013	19.6	19.1	15.0	12.3	8.5	3.7	5.2	14.6	6.0	0.8	1.1	26.6	12.8	3.9	5.4	70.3	61.1	28.8	39.9
2014	19.4	19.4	16.0	13.2	8.5	2.5	3.5	16.4	6.2	0.7	1.0	25.6	12.5	2.4	3.4	70.5	63.4	22.9	32.6
2015			12.0			2.3	3.3			0.6	0.8			2.5	3.5			24.3	34.8
2016			11.2			2.9	4.0			1.2	1.7			2.2	3.0			26.8	36.9
Mean (SD) of annual % rates	19.0 (0.89)	19.2 (0.56)	13.6 (2.30)	9.6 (1.77)	9.0 (0.87)	2.8 (0.64)	4.0 (0.84)	9.8 (3.32)	6.4 (0.66)	0.8 (0.28)	1.2 (0.38)	28.7 (2.08)	11.4 (1.41)	2.8 (0.78)	3.9 (1.06)	68.6 (2.12)	57.9 (4.01)	25.7 (2.60)	36.0 (3.10)

*BC HC* British Columbia RAI-HC data from the Home Care Reporting System, *CHA* Community Health Assessments from Ontario; CHA (All): Results from all CHA assessments, including those without a completed functional supplement module, which contains this variable; CHA (FS): Results from only CHA assessments with a completed functional supplement module which contains this variable *ON HC* Ontario RAI-HC data from the Ontario Association of Community Care Access Centres, *OT* Occupational Therapy, *PSW* Personal Support Worker services, *PT* Physical Therapy

**Table 6:** Trends in resource intensity

	RUG-III/HC Reduced Physical Function (%)		RUG-III/HC Extensive Services (%)		Mean CMI (Formal and informal)	
Year	ON HC	BC HC	ON HC	BC HC	ON HC	BC HC
2003	56.9		2.0		0.95	
2004	56.2		2.0		0.96	
2005	56.1		2.0		0.95	
2006	55.4		2.0		0.95	
2007	54.1		2.1		0.95	
2008	56.6	47.3	2.0	2.7	0.94	1.14
2009	56.5	48.1	2.0	2.7	0.95	1.13
2010	53.6	47.7	2.3	2.8	0.99	1.12
2011	50.5	46.7	2.5	2.9	1.03	1.13
2012	48.8	45.3	2.6	3.1	1.06	1.16
2013	46.5	45.3	2.8	3.2	1.10	1.16
2014	45.6	45.6	2.9	3.1	1.11	1.16
Mean (SD) of annual % rates or means	53.1 (4.13)	46.6 (1.17)	2.3 (0.34)	2.9 (0.19)	1.00 (0.06)	1.14 (0.02)

*BC HC* British Columbia RAI-HC data from the Home Care Reporting System, *CMI* Case Mix Index, *ON HC* Ontario RAI-HC data from the Ontario Association of Community Care Access Centres, *RUG-III/HC* Resource Utilization Groups – Home Care

Ontario HC clients were generally more likely to receive any physical therapy, occupational therapy, nursing and personal support worker services (PSW) than their counterparts in BC or in Ontario CSS. There was increased access to physical therapy and occupational therapy in Ontario over time, but lower rates of receiving nursing. On the other hand, there was a modest decline in BC clients’ access to physical therapy but increased access to nursing and PSW services.

There was a decrease of about 11% in the percentage of Ontario HC clients falling in the lowest RUG-III/HC categories (reduced physical function), while only a slight decrease in the percentage of BC clients in those groups. In both provinces, the percentages in the extensive services categories were stable between 2 to 3% of cases. These differences in RUG-III/HC groups are reflected in the increased resource intensity. In BC, mean CMI values were generally higher than in Ontario, and increased from 2008 to 2014, ranging from 1.12 to 1.16. CMI values are not calculated for CSS clients. These changes are relatively important, because the increased CMI from 0.95 to 1.11 reflects a relative increase of overall resource intensity of about 16.8% in Ontario home care clients. The corresponding increase in BC amounts to about a 4% increase in resource intensity compared with their 2008 population.

### Trends in convergent validity

Table 7 reports on trends in indicators of convergent validity over time by examining the associations between ADLH and CPS; IADL capacity and CPS; IADL performance and CPS; Pain and DRS; and Pain and CHESS. The correlations between these variables are investigated to assess the magnitude and direction of the associations and the trends are examined to determine whether the associations are stable over time.

**Table 7:** Trends in convergent validity (Pearson’s r correlation)

Year	ADLH & CPS			IADL (cap) & CPS			IADL (perf) & CPS			Pain and DRS			CHESS & Pain		
	ON HC	BC HC	CHA	ON HC	BC HC	CHA	ON HC	BC HC	CHA	ON HC	BC HC	CHA	ON HC	BC HC	CHA
2003	0.44			0.42			0.54			0.14			0.15		
2004	0.45			0.42			0.54			0.15			0.14		
2005	0.44			0.42			0.53			0.14			0.14		
2006	0.45			0.42			0.54			0.15			0.14		
2007	0.44			0.42			0.54			0.15			0.14		
2008	0.44	0.44		0.42	0.43		0.54	0.54		0.16	0.18		0.14	0.07	
2009	0.43	0.42		0.42	0.42		0.54	0.54		0.16	0.16		0.14	0.08	
2010	0.44	0.41		0.43	0.41		0.54	0.54		0.16	0.16		0.14	0.09	
2011	0.43	0.43		0.44	0.43		0.54	0.55		0.17	0.16		0.15	0.10	
2012	0.43	0.43		0.43	0.43		0.54	0.54		0.17	0.16		0.15	0.10	
2013	0.42	0.43	0.29	0.43	0.43	0.43	0.54	0.54	0.52	0.17	0.16	0.18	0.15	0.09	0.20
2014	0.43	0.43	0.25	0.43	0.44	0.40	0.53	0.55	0.51	0.18	0.16	0.18	0.16	0.08	0.20
2015			0.22			0.40			0.48			0.20			0.22
2016			0.26			0.41			0.49			0.17			0.20
Mean (SD) of annual R values	0.44 (0.01)	0.43 (0.01)	0.26 (0.03)	0.43 (0.01)	0.43 (0.01)	0.41 (0.01)	0.54 (0.00)	0.54 (0.00)	0.50 (0.02)	0.16 (0.01)	0.16 (0.01)	0.18 (0.01)	0.15 (0.01)	0.09 (0.01)	0.21 (0.01)

*ADLH* Activities of Daily Living Hierarchy scale, *BC HC* British Columbia RAI-HC data from the Home Care Reporting System, *CHA* Community Health Assessments from Ontario, *CHESS* Changes in Health, End-Stage Disease, Signs, and Symptoms Scale, *CPS* Cognitive Performance Scale, *DRS* Depression Rating Scale, *IADL (cap)* Instrumental Activities of Daily Living scale, *IADL (perf)* Instrumental Activities of Daily Living self-performance scale, *ON HC* Ontario RAI-HC data from the Ontario Association of Community Care Access Centres, *Pain* interRAI Pain Scale

A relatively stable, moderate positive correlation was observed between the ADLH and CPS, with values falling within a narrow range of 0.42 to 0.45 in the Ontario RAI-HC data (2003-2014) and 0.41 to 0.44 in the BC RAI-HC data (2008-2014). In the CHA data, a weaker positive correlation was observed, with r values falling between 0.22 and 0.29. The associations between IADL capacity and CPS were fairly consistent between provinces and stable over time in the RAI-HC data, with the r value ranging from 0.42 to 0.44 in Ontario and between 0.41 and 0.44 in BC throughout the study time period. The relationship between IADL capacity and CPS is similar in the CHA data, with the r value ranging from 0.40 to 0.43. The correlations between IADL self-performance and CPS behaved in a similar way. The r value held constant at 0.53 or 0.54 in Ontario and at 0.54 or 0.55 in BC throughout the study time period. The relationship between IADL self-capacity and CPS is only slightly lower in the CHA data, with r values falling between 0.48 and 0.52. The correlation between Pain and DRS was lower in magnitude, but remained positive and fairly stable, ranging from 0.14 to 0.18 in Ontario RAI-HC data and from 0.16 to 0.18 in BC RAI-HC data. This correlation was similar in the CHA data, ranging from 0.17 to 0.20. A somewhat weaker positive correlation was observed between Pain and CHESS in Ontario and BC HC clients, but values were quite stable over time ranging from 0.14 to 0.16 in the Ontario RAI-HC data and from 0.08 to 0.10 in the BC RAI-HC data. On the other hand, the association was slightly stronger in the CHA data with an r value ranging from 0.20 to 0.22. The convergent validity and quality of the data are supported by the stability of the correlations over time, indicating that the associations between the scales did not change dramatically.

### Trends in reliability

Trends in scale reliability are examined in Table 8 using Cronbach’s alpha to measure internal consistency. Cut-off points were set based on those cited in previous literature [1]. An alpha value of 0.70 or higher indicated acceptable reliability and an alpha value of 0.80 or higher indicated excellent reliability. Each of the scales exhibited acceptable or excellent reliability stable throughout the study period in Ontario RAI-HC data (from 2003 to 2014), BC RAI-HC data (from 2008 to 2014) and CSS CHA data (from 2013 to 2016). DRS had the lowest alpha values (range: ON, 0.72 to 0.74; BC, 0.74 to 0.76; CHA, 0.73-0.76) while the ADL Long Form scale had the highest alpha values (range: ON, 0.92 to 0.93; BC, 0.93 to 0.94; CHA, 0.92 to 0.93).

**Table 8:** Trends in reliability (Cronbach’s alpha)

year	IADL (perf)			IADL (cap)			DRS			ADL – Long Form		
	ON HC	BC HC	CHA	ON HC	BC HC	CHA	ON HC	BC HC	CHA	ON HC	BC HC	CHA
2003	0.87			0.87			0.74			0.92		
2004	0.87			0.87			0.73			0.93		
2005	0.87			0.87			0.73			0.93		
2006	0.87			0.87			0.72			0.93		
2007	0.87			0.87			0.72			0.93		
2008	0.87	0.89		0.87	0.87		0.72	0.76		0.93	0.93	
2009	0.87	0.89		0.87	0.87		0.72	0.74		0.93	0.93	
2010	0.87	0.88		0.87	0.86		0.72	0.75		0.92	0.93	
2011	0.87	0.88		0.87	0.87		0.72	0.74		0.92	0.93	
2012	0.87	0.88		0.86	0.86		0.73	0.74		0.92	0.94	
2013	0.87	0.88	0.83	0.85	0.87	0.88	0.73	0.75	0.76	0.92	0.93	0.93
2014	0.86	0.88	0.82	0.85	0.86	0.87	0.72	0.75	0.76	0.92	0.93	0.93
2015			0.82			0.86			0.74			0.92
2016			0.82			0.86			0.73			0.93
Mean (SD) of annual alpha values	0.87 (0.00)	0.88 (0.00)	0.82 (0.01)	0.87 (0.01)	0.87 (0.01)	0.87 (0.01)	0.73 (0.01)	0.75 (0.01)	0.75 (0.02)	0.93 (0.01)	0.93 (0.00)	0.93 (0.01)

*ADLH* Activities of Daily Living – Long Form scale, *BC HC* British Columbia RAI-HC data from the Home Care Reporting System, *CHA* Community Health Assessments from Ontario, *CPS* Cognitive Performance Scale, *DRS* Depression Rating Scale, *IADL cap* Instrumental Activities of Daily Living scale, *IADL perf* Instrumental Activities of Daily Living self-performance scale, *ON HC* Ontario RAI-HC data from the Ontario Association of Community Care Access Centres

### Trends in auto-population

Table 9 shows trends in indicators of potential auto-population based on the absence of change in particular sets of indicators from one assessment to the next. Informal hours of care was a variable that showed high rates of identical values from the first assessment in a year to the second (rates ranged from 50.6 to 68.7%). The general trend towards an increasing percentage of cases where there was no change over time may reflect an increased tendency to use auto-population in follow-up assessments. However, it is also possible that as care recipients become more complex and require more care, informal care providers reach a ceiling in the amount of care they can provide and the stability in the trends may reflect a reaching of this ceiling. Future research could further explore this possibility. Alternatively, the stability may reflect the fact that informal care providers generally provide the same amount of care each week and round the number of hours up or down when completing the assessment. In ADL self-performance items, and IADL (self-performance and capacity) items, there is also an overall trend towards increasing percentages of cases where there was no change in sets of indicators over time. However, the rates of no change in values were generally low and did not change very drastically from 2003 to 2013 (ADL items: increased by over 4%; IADL items combined: increased by over 11%). While it is possible that the stability in trends accurately reflects rates of auto-population, the stability may indicate a true absence of clinical change over time. Finally, in the set of mood items, there is a trend towards decreasing rates of no change from 2003 to 2014, from a maximum of 44.0% to 34.5%. Before excluding those who have a score of 0 at both time points, there were much higher rates of possible auto-population in each set of indicators and they decreased over time.

**Table 9:** Trends in auto-population

Year	Informal hours of care (combined)	ADL	IADL (both)	IADL (performance)	IADL (capacity)	Mood
2003	50.6	1.8	9.7	10.9	14.9	38.7
2004	58.8	2.5	11.9	13.1	16.5	41.4
2005	61.0	2.5	10.7	11.8	15.9	44.0
2006	65.5	2.7	10.6	12.0	15.5	42.3
2007	68.4	2.7	10.6	12.2	15.7	43.2
2008	68.7	2.7	10.9	12.3	16.2	43.2
2009	67.4	2.8	11.2	12.8	16.7	42.2
2010	64.4	2.9	12.6	14.5	19.8	37.2
2011	64.6	3.7	14.9	17.0	23.5	36.2
2012	64.5	4.3	17.2	19.3	26.9	35.1
2013	64.2	5.5	19.9	22.4	31.4	34.5
2014	65.3	6.0	20.8	23.3	31.9	34.6
Mean (SD) of annual % rates	63.3 (4.97)	3.4 (1.29)	13.4 (3.86)	15.1 (4.33)	20.4 (6.37)	39.4 (3.72)

*ADL – Long Form* Activities of Daily Living Long Form scale, *IADL (capacity)* Instrumental Activities of Daily Living scale, *IADL (performance)* Instrumental Activities of Daily Living self-performance scale

### Patterns of association in statistical indicators across provinces

**Fig. 1:**
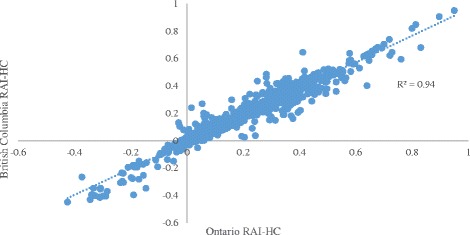
Association between statistical indicators obtained from the RAI-HC in Ontario and British Columbia

Figure 1 displays the results of analyses used to assess how well patterns of associations in RAI-HC data from Ontario compare to those obtained from BC based on the approach used in Hirdes and colleagues [1]. The analysis includes Pearson’s correlation coefficients for ADLH, IADL, CPS, MAPLe and hours of informal care with each other; Cronbach’s alpha values for IADL (performance), ADL Long Form and DRS; and Spearman’s Rank Sum correlations for several individual items. The points on the plot represent a comparison of the associations of over 180 various statistical tests between the two provinces. The high R^2^ value of 0.94 suggests that items in the RAI-HC behave in the same way in both provinces, which is an indication of good data quality.

**Fig. 2:**
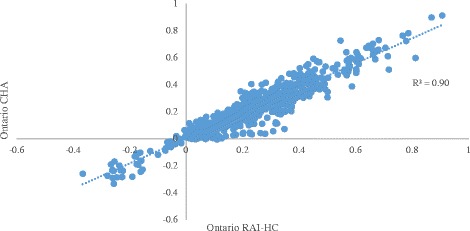
Association between statistical indicators obtained from the RAI-HC in Ontario and the CHA in Ontario

Figure 2 displays the results of the same analysis comparing Ontario RAI-HC data to CHA data. Similarly, the high R^2^ value of 0.90 suggests that items in the CSS sector behave in the same way as in the HC sector in Ontario, further lending support to conclusions of good data quality in CSS organizations.

## Discussion

Overall, the results of the quality of RAI-HC and CHA data analyses are positive, providing consistent evidence of good validity and reliability of data in home and community care in Ontario and BC. The results of the analyses describing population characteristics are unsurprising across the board, but they do suggest that there has been a notable change in the Ontario HC population over time. The BC HC population was somewhat more consistent in its composition. From the limited number of years available for CHA-assessed CSS clients, trends appear stable. However, it is possible that there is not a long enough period of time to discern strong trends. The lower percentages of moderate to severe cognitive impairment and possible depression in CSS clients compared to HC clients is to be expected. Clients with higher care needs within the CSS sector are likely also HC clients and would have had a RAI-HC assessment completed by the Community Care Access Centre rather than a CHA assessment.

The results indicated that HC clients in BC were more cognitively impaired than in Ontario although the percentage of cognitively impaired clients increased over time in the latter. There was also a higher proportion of clients with possible depression and CPS, DRS, MAPLe, ADLH, IADL capacity and self-performance scores of three or more, and a higher mean CMI in BC than in Ontario, stable over time. This discrepancy may be a reflection of differences in policy and eligibility for home care and long-term care services. For example, the trend towards increasing client complexity in Ontario is consistent with a broader policy shift towards discharging patients home from acute care settings prior to long-term care admission in the province**.** Trends in increased resource intensity in Ontario reflected the fact that the client population became considerably more complex over time. The total hours of informal care received were similar across provinces and were fairly stable over time suggesting families were providing consistent levels of support. This stability despite increasing client complexity in Ontario may reflect a ceiling effect, where informal care providers can only provider a certain level of care. Once reached, care provided by informal caregivers may reach a limit despite increasing care needs.

Generally, the RAI-HC data from Ontario and BC behave in a consistent manner, despite the changing and increasingly complex home care population in Ontario. The trends in internal consistency in the Ontario and BC RAI-HC data were stable, indicating good reliability. The alpha values measured in the RAI-HC data were generally consistent with those found in the evaluation of RAI-MDS 2.0 data quality, reported by Hirdes and colleagues [1]. Similarly, the trends in convergent validity in Ontario and BC RAI-HC data were stable over time, indicating good quality of data. The correlations between scales observed in both provinces are generally similar and are consistent with what was reported in the literature concerning the associations between the same scales in the RAI-MDS 2.0 data. In addition, the differences in magnitudes of associations between variables were consistent with what one would expect (e.g., the relationship between ADLH and cognition was stronger than the relationship between pain and health instability). The rates of potential auto-population found in the Ontario RAI-HC data are lower than the rates reported by Hirdes and colleagues [1] in the RAI-MDS 2.0 data. The difference may reflect the difference in care settings and patient characteristics or differences in the way the analyses were performed.

The results from the analyses of the CHA data indicate that the quality of the data matches the quality of RAI-HC data in most cases (e.g. convergent validity of both measures of IADLs & CPS; Pain and DRS). The very strong associations between the indicators examined in Fig. 1 suggest that the data from Ontario and BC RAI-HC assessments behave in a highly consistent manner across several tests of validity and reliability. Therefore, one may be confident in making comparisons between measures of needs or quality of care using RAI-HC assessment records from these two provinces. Similarly, strong associations between indicators examined in Fig. 2 suggest that the data from the HC and CSS sectors in Ontario behave consistently when evaluating reliability and validity.

This study is the first to examine evidence of reliability and validity of interRAI CHA assessments in Ontario, which are often done by community care staff with fewer professional credentials than case managers in Ontario and BC. The evidence reported here suggested that the data behave in a comparable manner to those obtained from case managers who are regulated health professionals. This also suggests that the educational aspects of implementing interRAI assessments using the item definitions and coding guidelines may be helpful in generating a consistent and standardized approach to measurement that can be employed by a variety of home and community care professionals.

Several limitations exist in this study, namely related to the CHA data available. The number of CHA assessments in the analyses is lower than the number of RAI-HC assessments and does not cover the same full time period because the CHA was implemented later and on a more limited scale. Further, CHA data are not obtained from the full client populations served by CSS organizations. Not all clients in every program have a CHA assessment completed, the practice policies are not transparent and they vary by organization and by program within organizations. The under-identification of hospital version assessments among the BC RAI-HC data is a second limitation of the study.

## Conclusions

The associations within and between scales were generally stable and consistent across provinces and sectors. This indicates good data quality, despite the challenges associated with doing assessments in the community, combined with changes toward increased complexity of home care clients, at least in Ontario. A major strength of this study was the sheer number of RAI-HC assessments included, from both Ontario and BC. The present study demonstrated that the statistical methods used to evaluate the quality of RAI-MDS 2.0 data in the long-term and continuing care settings may be used to evaluate the quality of RAI-HC data in the home care sector. Further, the results support the use of the same analyses to analyze the quality of CHA data within the CSS sector. High quality data and information is vital at both a clinical practice and policy level when used for decision-making to improve quality of care and enhance quality of life for individuals who rely on home and community care services.

## Additional file

Additional file 1: Number of assessments included for each setting and province by year (N). In order to reduce the size of the tables of results, a summary table was created to show the number of assessments included in the analyses for each setting and province by year. (DOCX 14 kb)

## Abbreviations

ADL: Activities of Daily Living Long-Form
ADLH: Activities of Daily Living Hierarchy
BC: British Columbia
CHA: Community Health Assessment
CHESS: Changes in Health, End-stage Diseases, Signs, and Symptoms Scale
CMI: Case Mix Index
CPS: Cognitive Performance Scale
CSS: Community support services
DRS: Depression Rating Scale
HC: Home care
IADL: Instrumental Activities of Daily Living
MAPLe: Method for Assigning Priority Levels
MMSE: Mini-Mental State Exam
ORE: Office of Research Ethics
PSW: Personal support worker
RAI-HC: Resident Assessment Instrument – Home Care
RAI-MDS 2.0: Resident Assessment Instrument – Minimum Dataset 2.0
