# Supplementary material for: Evaluation of data quality of interRAI assessments in home and community care
Source: BMC Med Inform Decis Mak. 2017 Oct 30;17:150. doi: 10.1186/s12911-017-0547-9 (PMC5663080; doi:10.1186/s12911-017-0547-9)
Supplement: Additional file 1: — Number of assessments included for each setting and province by year (N). In order to reduce the size of the tables of results, a summary table was created to show the number of assessments included in the analyses for each setting and province by year. (DOCX 14 kb) [file 12911_2017_547_MOESM1_ESM.docx]

|  | **ON HC** | **BC HC** | **CHA (All)** | **CHA (FS)** |
| --- | --- | --- | --- | --- |
| **2003** | 59634 |  |  |  |
| **2004** | 117379 |  |  |  |
| **2005** | 136604 |  |  |  |
| **2006** | 144778 |  |  |  |
| **2007** | 147323 |  |  |  |
| **2008** | 151809 | 12879 |  |  |
| **2009** | 157080 | 21049 |  |  |
| **2010** | 155857 | 33353 |  |  |
| **2011** | 162496 | 35369 |  |  |
| **2012** | 166023 | 32823 |  |  |
| **2013** | 166000 | 35039 | 7157 | 5165 |
| **2014** | 178235 | 38223 | 13886 | 9776 |
| **2015** |  |  | 16330 | 11397 |
| **2016** |  |  | 7806 | 5668 |
| **Total** | 1743218 | 208735 | 45179 | 32006 |
| BC HC = British Columbia RAI-HC data from the Home Care Reporting System; CHA = Community Health Assessments from Ontario; CHA (All): Results from all CHA assessments, including those without a completed functional supplement module, which contains this variable; CHA (FS): Results from only CHA assessments with a completed functional supplement module which contains this variable; ON HC = Ontario RAI-HC data from the Ontario Association of Community Care Access Centres | | | | |
